# Flexible human serum albumin nanocapsules to enhance drug delivery and cellular uptake for photodynamic/chemo cancer therapy

**DOI:** 10.1039/d2ra06859a

**Published:** 2023-02-14

**Authors:** Wen Kang, Yuyuan Shi, Zhenlu Yang, Xindao Yin, Ying Zhao, Lixing Weng, Zhaogang Teng

**Affiliations:** a Department of Radiology, Nanjing First Hospital, Nanjing Medical University Nanjing 210006 P. R. China zhaoyingmed@163.com; b College of Geography and Biological Information, Nanjing University of Posts and Telecommunications Nanjing 210023 P. R. China lxweng@njupt.edu.cn; c Department of Radiology, Guizhou Provincial People's Hospital Guiyang Guizhou P. R. China; d Key Laboratory for Organic Electronics & Information Displays and Jiangsu Key Laboratory for Biosensors, Institute of Advanced Materials, Jiangsu National Synergetic Innovation Centre for Advanced Materials, Nanjing University of Posts and Telecommunications (NJUPT) Nanjing 210023 P. R. China tzg@fudan.edu.cn

## Abstract

As a non-invasive cancer treatment, photodynamic therapy (PDT) has great applications in superficial tumors because of its high selectivity and low cumulative toxicity. However, the poor tumor-targeting ability and short blood circulation time of conventional photosensitizers (PSs) limit the efficacy of PDT to some extent. In this study, we synthesized flexible hollow human serum albumin (HHSA) and loaded photosensitizer Chlorin e6 (Ce6) and the chemotherapeutic drug Doxorubicin (DOX) for synergistic cancer therapy. HHSA can enhance drug delivery and cellular uptake through targeting gp60 and SPARC receptors and unique flexible hollow structures. The TEM images show that HHSA possesses distinct flexible hollow structures, as well as good monodispersity and deformability. After loading Ce6 and DOX, HHSA@Ce6-DOX displays better therapeutic effects than HHSA@DOX on the growth of 4T1 breast cancers without irradiation. Remarkably, it has a significantly higher therapeutic effect (relative cell activity: 45% *vs.* 74%) than HHSA@Ce6 under 660 nm irradiation. Furthermore, the excellent biocompatibility of HHSA@Ce6-DOX has been proved both *in vitro* and *in vivo*, indicating that it has a promising future in synergistic tumor treatments.

## Abstract 2

In this study, we synthesized flexible hollow human serum albumin (HHSA), which can enhance drug delivery and cellular uptake, and loaded in photosensitizer Chlorin e6 (Ce6) and chemotherapeutic drug Doxorubicin (DOX) for synergistic cancer therapy.

## Graphical abstract

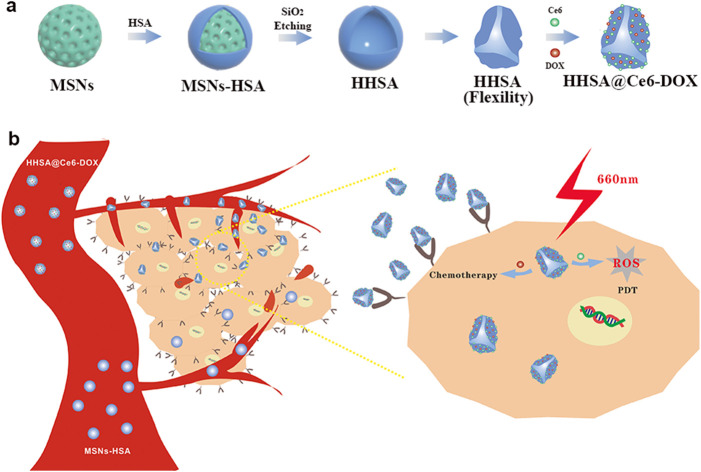

## 1. Introduction

Photodynamic therapy (PDT), as a non-invasive cancer treatment, has the advantages of high temporal and spatial selectivity, repeatability, and low cumulative toxicity, which has attracted the extensive attention of researchers.^1^ When the photosensitizers (PSs) absorb the energy from a certain wavelength of light, they will transform the ground state (S0) to the first excited state (S1). A more stable triple excited state (T1) will be formed *via* a process called “intersystem crossing” because S1 is unstable. The PSs in T1 collide with the surrounding ground oxygen molecule (^3^O_2_) and carry out energy transfer to produce singlet oxygen (^1^O_2_), which is a photodynamic type II process. The process of hydrogen abstraction or electron transfer occurs between excited PSs of type I, which will be accompanied by the formation of radical ions. These radical ions can react with ^3^O_2_ to generate reactive oxygen species (ROS), such as superoxide anion (O^2−^˙), hydrogen peroxide (H_2_O_2_), and hydroxyl radical (OH˙). At present, most of the second-generation PSs are porphyrin and chlorin structures which are excited *via* red light between 650 nm and 700 nm.^2,3^ However, the conventional photosensitizers (PSs) have some deficiencies, including poor tumor-targeting ability and short blood circulation time.^4^ Some novel drug delivery carriers can be designed to solve these problems. Human serum albumin (HSA), the most abundant plasma protein in the body, is an important transporter with good biocompatibility and degradability.^5^ HSA can be combined with hydrophobic drugs in a reversible way to assist their transport in the human body and can effectively target tumor cells through gp60 and SPARC receptors-mediated endocytosis to enhance the uptake of tumor cells.^6^ Therefore, HSA has the potential as a tumor-targeted drug delivery carrier.

At present, many studies have shown that compared with rigid nanomaterials of the same material, flexible nanomaterials have longer blood circulation time, higher cell uptake rate and less internalization, and phagocytosis by macrophages or other immune cells, which is conducive to the accumulation of nanomaterials in tumor sites.^7,8^ Xu *et al.* proved that the flexible HSA-based nanoprobes can improve the cell uptake efficiency and lengthen residence time *in vivo*, which helps to improve the curative effect of nanodrugs.^9^ The stiffness adjustment of nanoparticles based on HSA provides a new way to improve the cellular uptake of PSs and other hydrophobic drugs.

Moreover, individual PDT may trigger treatment escape pathways due to the damage to the tumor microenvironment and the induction of vascular endothelial growth factor (VEGF) in local tumor tissue.^10,11^ Although PDT is effective for solid tumors, it can only kill tumor cells in the irradiation area and is not suitable for the treatment of metastatic tumors. Therefore, it is necessary to design a suitable nanoplatform for synergistic therapies and combine PDT with other tumor therapies to overcome these shortcomings and achieve more efficient treatment effects.

In this work, we designed a nanocapsule HHSA@Ce6-DOX based on flexible hollow human serum albumin nanocapsule (HHSA) and loaded photosensitizer Chlorin e6 (Ce6) and chemotherapeutic drug Doxorubicin (DOX) for synergistic cancer therapy. HHSA nanocapsules have a uniform size, good dispersion and a hollow structure. The hollow structure can decrease the stiffness of nanocarriers and achieve an efficient drug loading rate. The flexible and tumor-targeting characteristic of HHSA is conducive to enhancing drug delivery and cellular uptake *via* prolonging the circulation time, reducing the absorption of the immune system and combining with receptors on the tumor cells. Meanwhile, the experimental results showed that, compared with a single treatment, HHSA@Ce6-DOX can achieve better curative effect *via* synergistic therapy.

## 2. Materials and methods

### 2.1 Materials

Cetyltrimethylammonium bromide (CTAB), tetraethoxysilane (TEOS), anhydrous ethanol, concentraethyl hydrochloric acid (HCl, 37 wt%), glutaraldehyde (GA, 25%), and hydrofluoric acid (HF, 48 wt% in H_2_O) were purchased from Sinopharm Chemical Reagent Co., Ltd (Shanghai, China). *N*,*N*-Dimethylformamide (DMF), chlorin e6 (Ce6), 2′,7′-dichlorofluorescin diacetate (DCFH-DA), poly(ethylene imine) (PEI, branched, Mw: 25 000), and human serum albumin (HSA) were obtained from Sigma-Aldrich (St. Louis, MO, USA). Singlet oxygen sensor green (SOSG) was purchased from Thermo (USA). *N*-hydroxysulfosuccinimide (NHS), *N*-(3-dimethylaminopropyl)-*N*′-ethylcarbodiimide hydrochloride (EDC) were acquired from Shanghai Aladdin Bio-Chem Technology Co., Ltd (Shanghai, China). Ammonia aqueous solution (NH_3_·H_2_O, 25 wt%) was bought from Nanjing Chemical Reagent CO., Ltd (Nanjing, China). Phosphate buffer saline (PBS), Roswell Park Memorial Institute (RPMI) 1640 medium, Dulbecco's Modified Eagle Medium (DMEM), Trypsin–EDTA (0.25%) and 3-(4,5-dimethylthiazol-2-yl)-2,5-diphenyltetrazolium bromide (MTT) were purchased from Nanjing Keygen Biotech. Co., Ltd (Nanjing, China). Fetal bovine serum (FBS) was purchased from Procell Life Science&Technology Co., Ltd (Wuhan, China). Doxorubicin (DOX) was purchased from Beijing Huafeng United Technology Co. Ltd (Beijing, China). The water used in all experiments is deionized water with a resistivity of 18 MΩ cm.

### 2.2 Preparation of mesoporous silica nanoparticles (MSNs)

MSNs were prepared by a modified surfactant-directed sol–gel method in which cetyltrimethylammonium bromide (CTAB) was used as a template and tetraethoxysilane (TEOS) provided silicon element. First, 0.16 g CTAB was dissolved in a mixed solution containing 30 mL ethanol, 75 mL water and 0.5 mL concentrated ammonia aqueous. Then 0.5 mL TEOS was added into the above solution with a 35 °C water bath. The white suspension (CTAB-MSNs) was obtained after being stirred (500 rpm, 3 h) and centrifuged (10 000 rpm, 10 min) sequentially, and the suspension was washed with ethanol 3 times. Finally, they were preserved in 10 mL ethanol solution. To remove the surfactant CTAB, CTAB-MSNs were dispersed in 200 mL ethanol solution containing 400 μL concentrated hydrochloric acid and the mixture was stirred in a 60 °C water bath (500 rpm). The above process was repeated three times to completely remove CTAB. The obtained product was washed twice with ethanol and then twice with water. Finally, MSNs without CTAB were dispersed in 12.5 mL of deionized water.

### 2.3 Preparation of flexible hollow HSA nanocapsules (HHSA)

4 mL MSNs aqueous solution was mixed with 20 mL Poly(ethylene imine) (PEI) aqueous solution (1 mg mL^−1^). The mixture solution was stood for 20 min and was washed with water 3 times to obtain PEI modified MSNs (MSNs-PEI) which was dispersed in 10 mL deionized water. Then, 10 mL MSNs-PEI aqueous solution and 10 mL GA aqueous solution were mixed and shaken at room temperature (200 times per min, 12 h). The shaken product was washed 3 times with deionized water to obtain MSNs-PEI-GA. 10 mL HSA aqueous solution (4 mg mL^−1^) was added to MSNs-PEI-GA aqueous solution and shaken to form MSNs-HSA (200 times per min, 12 h). The product was washed 3 times and dispersed in 10 mL of deionized water. Finally, HF solution (40 wt%) was added to the MSNs-HSA aqueous solution and blown with a pipette gun for 1 min to remove MSNs to obtain HHSA.

### 2.4 Preparation of monotherapy nanocapsules (HHSA@Ce6, HHSA@DOX)

10 mg Ce6, 5 mg EDC and 5 mg NHS were dissolved in 1.5 L DMF, and the mixture was shaken to activate the carboxyl group of Ce6 (200 times per min, 3 h, dark). Subsequently, 10 mL HHSA aqueous solution was added to the mixture and shaken the mixed solution (200 times per min, 12 h, dark). The product was collected by centrifugation and washed three times with DMF to obtain HHSA@Ce6.

5 mL HHSA aqueous solution and 5 mL DOX solution were mixed and shaken (200 times per min, 18 h, dark) and the mixture was centrifuged and washed to obtain HHSA@DOX.

### 2.5 Preparation of synergistic therapeutic nanocapsules (HHSA@Ce6-DOX)

5 mL HHSA@Ce6 aqueous solution and 5 mL DOX solution (1 mg mL^−1^) were mixed and shaken (200 times per min, 18 h, dark). The product was collected by centrifugation and washed 3 times.

### 2.6 Characterization of nanoparticles

TEM images were taken *via* an HT7700 microscope (Hitachi, Tokyo, Japan) at 100 kV. The Brookhaven analyser (Brookhaven Instruments Co., Holtsville, NY, USA) was used to detect the zeta potential and hydrodynamic sizes. FT-IR spectra were obtained by the Nicolet Nexus 870 spectrometer (Nicolet Instruments Inc., Madison, WI, USA). UV-vis spectra were measured on a Lambda 35 UV-vis spectrophotometer (PerkinElmer, Inc., Waltham, MA, USA).

### 2.7 Evaluation of cytotoxicity

4T1 cells were cultured in the 96 well plates (15 × 10^4^ cells per well) for 24 h and 100 μL fresh complete medium containing different concentrations (0, 12.5, 25, 50, 100, 200 μg mL^−1^ HHSA equiv.) of HHSA, HHSA@DOX, HHSA@Ce6 and HHSA@Ce6-DOX were added, respectively. The 96 well plates were washed twice and added 50 μL MTT to each well after 24 h and continued to culture for 4 h. After removing the upper liquid, the purple crystal was dissolved with DMSO. The groups without nanoparticles (0 μg mL^−1^) were used as the control groups. The absorbance of each well at 490 nm was recorded with a multifunctional microplate reader (Tecan Infinite PRO TWIN 200, Switzerland).

### 2.8 Generation of ROS after 660 nm laser irradiation

SOSG probes were used to detect the generation of ROS of HHSA@Ce6-DOX after irradiation. The experiment was divided into 3 groups: (1). HHSA@Ce6-DOX + Laser (0.5 W cm^−2^) group, (2). HHSA@Ce6-DOX + Laser (1 W cm^−2^) group, (3). HHSA@Ce6-DOX + Laser (1.5 W cm^−2^) group (1 μg mL^−1^ Ce6 equiv.), and each group was set with 3 holes. HHSA@Ce6-DOX nanomaterials (1 μg mL^−1^ Ce6 equiv., 100 μL) and SOSG probes (50 μM, 50 μL) were mixed in the 96 well plates with irradiation for 0, 1, 3, 5 and 10 min, the fluorescence intensity of the mixture was detected at Ex/Em = 504/525 nm *via* a multifunctional microplate reader.

### 2.9 Evaluation of photodynamic therapy *in vitro*

4T1 cells and 293T cells were seeded in the 96 well plates (15 × 10^4^ cells per well) for 24 h, respectively. After the cells had adhered to the wall, 100 μL complete medium containing HHSA@Ce6 and HHSA@Ce6 -DOX (0.625 μg mL^−1^ Ce6 equiv.) was added, and the cell continued to cultivate for 24 h. Each well was irradiated with a 660 nm laser (1 W cm^−2^, 5 min) after washing twice. Then, each well was added 50 μL MTT. The absorbance of each well at 490 nm was recorded with a multifunctional microplate reader according to the above-mentioned method. The groups without nanoparticles (0 μg mL^−1^) were set as the control group.

### 2.10 Detection of ROS in the tumor cells

After 4T1 cells were seeded in the 96 well plates (15 × 10^4^ cells per well) for 24 h, HHSA@Ce6 and HHSA@Ce6-DOX (Ce6 equivalent to 0.625 μg mL^−1^, 100 μL) were added and incubated with 4T1 cells for 24 h. Next, each well was washed with PBS twice. 100 μL 1640 RPMI medium without FBS containing 10 μM DCFH-DA was added and then incubated for 20 min at 37 °C in the dark. After being irradiated by a 660 nm laser for 5 min (1 W cm^–2^), each well was washed with serum-free 1640 RPMI medium three times. The generation of intracellular ROS was measured by a microplate reader to detect the fluorescence intensity (Ex/Em = 488 nm/529 nm) between different groups.

### 2.11 LIVE/DEAD viability assay

4T1 cells were cultured in the 96 well plates (15 × 10^4^ cells per well) for 24 h and different nanomaterial solutions samples (Ce6 equivalent to 0.625 μg mL^−1^) were added into the 96 well plates and the cells were continued to cultivate for 24 h. 4T1 cells in different groups after being irradiated with 660 nm laser (1 W cm^−2^, 5 min) were strained by the live/dead kit. The HHSA@Ce6 group and HHSA@Ce6-DOX group without irradiation were set as the control groups. The distribution of live cells (green fluorescence) and dead cells (red fluorescence) was observed by a fluorescence microscope (Nikon, Japan).

### 2.12 *In vivo* biocompatibility

The healthy BALB/c mice (5 weeks old, 18–20 g) were randomly divided into the PBS group and the HHSA@Ce6-DOX (Ce6 equivalent to 10 μg mL^−1^) group. Each group had 3 mice. Different solutions (100 μL) were injected through the tail vein and all mice were sacrificed after 24 h. Then, tissue sections of major organs of different groups were prepared and stained with H&E to evaluate the systemic toxicity of HHSA@Ce6-DOX.

### 2.13 The tumor model

Female Balb/c mice (5 weeks, about 15–18 g) were provided by the Experimental Animal Center of Nanjing First Hospital of Nanjing Medical University. 4T1 cells suspension (2 × 10^7^/mL, 100 μL) were subcutaneously injected at the root of the right thigh of each mouse. When the tumor grew to about 250 mm^3^, 4T1 tumor-bearing mice were successfully constructed.

### 2.14 The photodynamic/chemo therapy *in vivo*

4T1 tumor-bearing mice were randomly divided into 3 groups (3 mice per group) and conducted the following treatments: (1) PBS; (2) HHSA@Ce6; (3) HHSA@Ce6-DOX. HHSA@Ce6 and HHSA@Ce6-DOX nanomaterials (Ce6 equivalent to 10 μg mL^−1^, 100 μL) or PBS (100 μL) were injected *via* tail vein, and the tumors of these mice were irradiated by a 660 nm laser (1 W cm^−2^) for 3 min after 24 hours. Tumor volume and body weight were observed and recorded every other day for 14 days. The tumor volume was calculated according to the following formula:Tumor volume *V* (mm^3^) = *d*^2^ × *D*/2*d* is the shortest diameter of the tumor and *D* is the longest diameter.

## 3. Results

### 3.1 Preparation and characterization of HHSA@Ce6-DOX

**Scheme 1:**
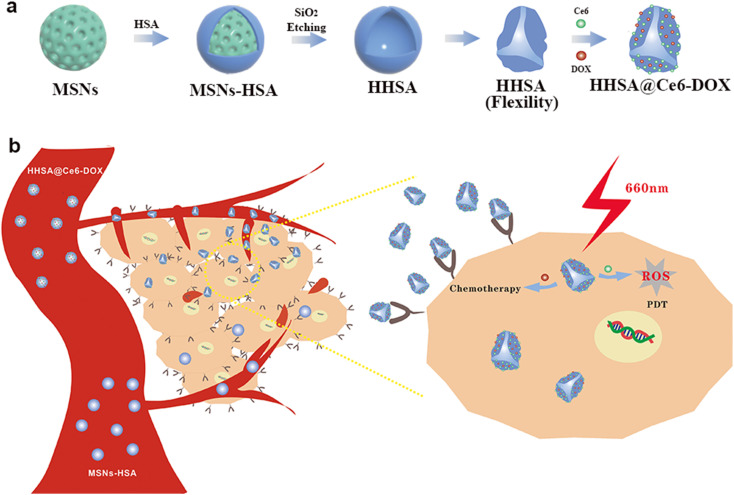
(a) Schematic illustration of the synthesis of HHSA and HHSA@Ce6-DOX. (b) The anti-tumor mechanism of HHSA@Ce6-DOX.

The complete synthesis process of flexible hollow HHSA@Ce6-DOX is illustrated in Scheme 1. They were synthesized by core-assisted protein-coating route. Firstly, mesoporous silica nanoparticles (MSNs), hard core template in the subsequent synthesis process, were prepared by a modified surfactant-directed sol–gel method which used CTAB as template and TEOS as silicon source.^12–14^ Poly(ethylene imine) (PEI) was adsorbed on the surface of MSNs *via* electrostatic interaction, and further modified with glutaraldehyde (GA) by condensation reaction of aldehyde and amino group. Next, the condensation reaction of amino and carboxyl groups made HSA connected with GA to produce core–shell MSNs-HSA nanospheres. Finally, the MSNs core of MSNs-HSA was etched with hydrofluoric acid (HF) to obtain flexible HHSA nanocapsules. To construct synergistic therapeutic nanoplatforms, photosensitizer Ce6 was combined with HHSA *via* amide reaction to obtain HHSA@Ce6 nanocapsules and the anticancer drug DOX self-assembled with HHSA@Ce6 to fabricate HHSA@Ce6-DOX nanocapsules. In addition, DOX can be directly loaded into the HHSA to produce HHSA@DOX.

**Fig. 1:**
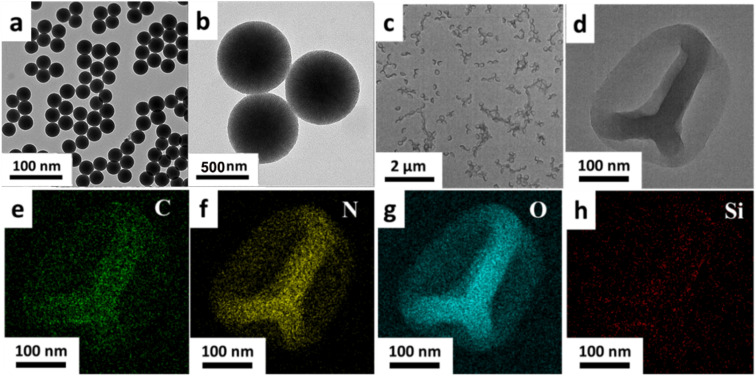
The TEM images of MSNs (a and b) and HHSA (c and d). The distribution images of C (e), N (f), O (g) and Si (h) elements in the HHSA.

The morphology characteristics of the MSNs and HHSA were characterized by the transmission electron microscope (TEM). MSNs had spherical morphology, uniform size and good dispersion (Fig. 1a and b). The TEM image of HHSA showed that the protein capsule was hollow and wrinkled on the surface, which gave them flexibility and deformability (Fig. 1c and d). Moreover, the element distribution image of HHSA showed that C, N and O were evenly distributed in the shell, and there is no obvious signal of Si element. These results further indicated that the hard core of MSNs-HSA has been successfully etched to form flexible hollow structures (Fig. 1e–h).

**Fig. 2:**
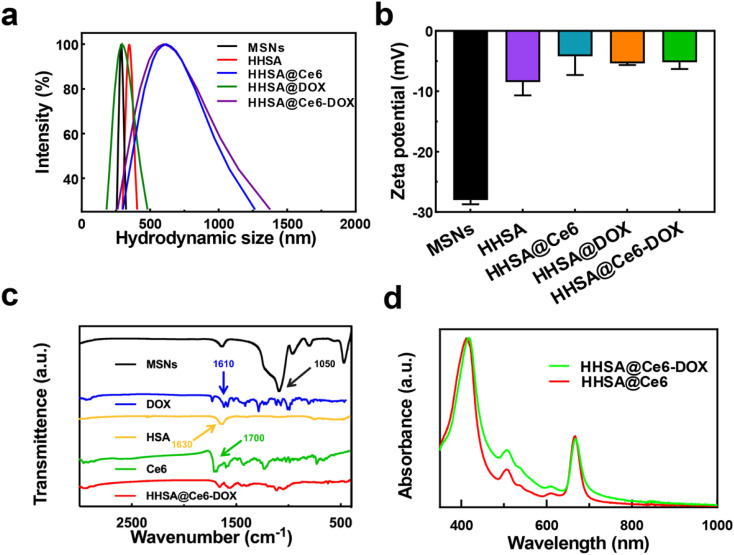
(a) The DLS diameters of MSNs, HHSA, HHSA@Ce6, HHSA@DOX and HHSA@Ce6-DOX. (b) The Zeta potential of MSNs, HHSA, HHSA@Ce6, HHSA@DOX and HHSA@Ce6-DOX. (c) The FT-IR spectrums of MSNs, DOX, HSA, Ce6, and HHSA@Ce6-DOX. (d) The UV-vis spectra of HHSA@Ce6 and HHSA@Ce6-DOX.

Dynamic light scattering (DLS) data display that the hydrodynamic diameters of MSNs, HHSA, HHSA@DOX, HHSA@Ce6 and HHSA@Ce6-DOX were about 280 nm, 350 nm, 320 nm, 615 nm, and 600 nm, respectively (Fig. 2a). MSNs, HHSA and HHSA@DOX had better dispersibilities due to smaller diameters. The sizes of HHSA@Ce6 and HHSA@Ce6-DOX were larger, and the dispersibility of them was poorer, owing to hydrophobic Ce6 with poor water dispersion combined with protein capsules and affected the dispersibility of protein capsules.^15^ The low dispersion makes the nanoparticles easy to agglomerate in the solution and reduces the bioavailability and the circulation time of the nanocapsules, thus affecting the therapeutic effect.^16^ The results of zeta potential showed that the synthesized material had the negative surface charge. The potential of MSNs, HHSA, HHSA@Ce6, HHSA@DOX and HHSA@Ce6-DOX were −28.00 ± 0.71 mV, −8.47 ± 2.09 mV, −4.21 ± 2.97 mV, −5.38 ± 0.30 mV and −5.18 ± 1.08 mV, respectively (Fig. 2b). Fourier transform infrared spectroscopy (FT-IR) showed that HHSA@Ce6-DOX had no characteristic absorption peak representing the Si–O–Si bond in MSNs at 1050 cm^−1^, indicating that the internal MSNs core had been successfully etched, and the hollow structure had been formed (Fig. 2c). FT-IR of HHSA@Ce6-DOX displayed there were two absorption peaks at 1600–1700 cm^−1^. This may be due to the coincidence of the absorption peaks of DOX, Ce6 and HSA at this band. To confirm the loading of Ce6 and DOX, the UV-vis spectra of HHSA@Ce6 and HHSA@Ce6-DOX were measured. The characteristic absorption peaks of Ce6, which are near 660 nm and 400 nm, appeared in the UV-vis spectra of HHSA@Ce6 and HHSA@Ce6-DOX, indicating that Ce6 is successfully loaded into HHSA@Ce6 and HHSA@Ce6-DOX (Fig. 2d). In addition, the absorption curve of HHSA@Ce6-DOX was higher than HHSA@Ce6 near the characteristic absorption peak of DOX—500 nm, demonstrating that DOX was successfully loaded into HHSA@Ce6-DOX.

### 3.2 *In vitro* toxicity test

**Fig. 3:**
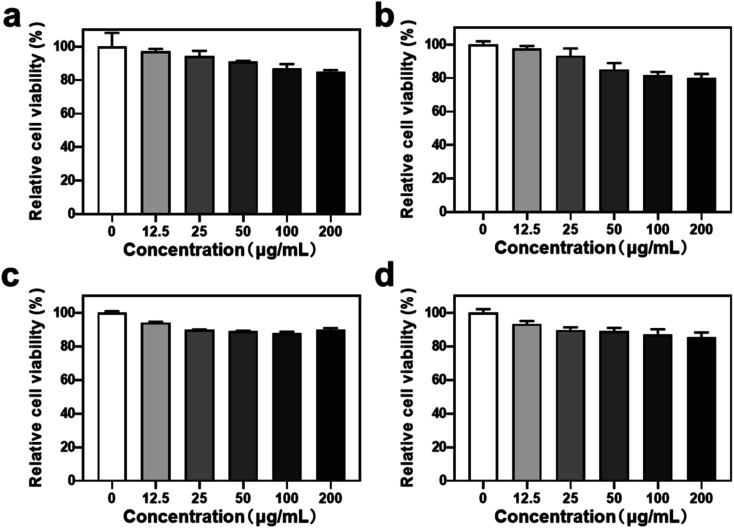
The relative cell activity of 4T1 cells treated with different nanodrugs for different times. (a) HHSA, 12 h; (b) HHSA, 24 h; (c) HHSA@Ce6, 12 h; (d) HHSA@Ce6, 24 h. The concentration of different nanocapsules was calculated according to the concentration of HHSA.

To evaluate the biomedical application potential of HHSA@Ce6-DOX, the MTT assay was used to detect the cytotoxicity of HHSA and HHSA@Ce6 in 4T1 breast cancer cells. The results showed that the relative cell viability exceeded 80% after incubation with 0–200 μg mL^−1^ (calculated according to the concentration of HHSA) HHSA and HHSA@Ce6 for 12 or 24 h, which confirmed that HHSA and HHSA@Ce6 had good biocompatibility (Fig. 3). Since HSA is a kind of endogenous protein, it did little damage to cells. At the same time, Ce6 had low toxicity without the irradiation of the laser. When the concentration reaches 200 μg mL^−1^, the relative cell viability in HHSA group was about 5% lower than that of HHSA@Ce6 group after incubating for 24 h. This is because, under the same quality, the system of HHSA without drug loading contained more nanoparticles than that of HHSA@Ce6. The normal growth of cells was affected by more nanocapsules, making the cell activity of HHSA group lower.

### 3.3 *In vitro* chemotherapy

**Fig. 4:**
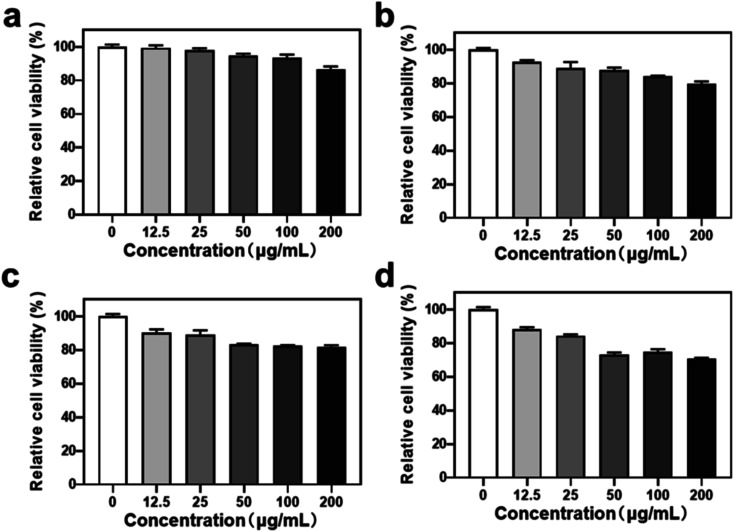
The relative cell viability of 4T1 cells treated with different nanocapsules for different times. (a) HHSA@DOX, 12 h; (b) HHSA@DOX, 24 h; (c) HHSA@Ce6-DOX, 12 h; (d) HHSA@Ce6-DOX, 24 h. The concentration of different nanocapsules was calculated according to the concentration of HHSA.

To assess the synergistic therapeutic effects of HHSA@Ce6-DOX, the chemotherapeutic effects of HHSA@DOX and HHSA@Ce6-DOX were first compared in 4T1 tumor cells *via* MTT assay. The results show that when 50 μg mL^−1^ HHSA@Ce6-DOX was incubated with 4T1 cells for 24 h, the relative cell viability decreased to about 75%, and the relative cell viability further decreased to about 70% when the concentration increased to 200 μg mL^−1^. Meanwhile, 200 μg mL^−1^ HHSA@DOX killed only about 20% 4T1 cells after 24 h (Fig. 4). As can be seen from the above results, the anti-cancer effect of HHSA@Ce6-DOX is better than that of HHSA@DOX. This may be because HHSA protein capsule has poor ability to bind with DOX resulting in low DOX loading. The hydrophobic Ce6 in the HHSA@Ce6-DOX nanocapsules could increase the drug loading of DOX through π–π stacking and hydrophobic interactions.^17^

### 3.4 ROS generation after irradiation

**Fig. 5:**
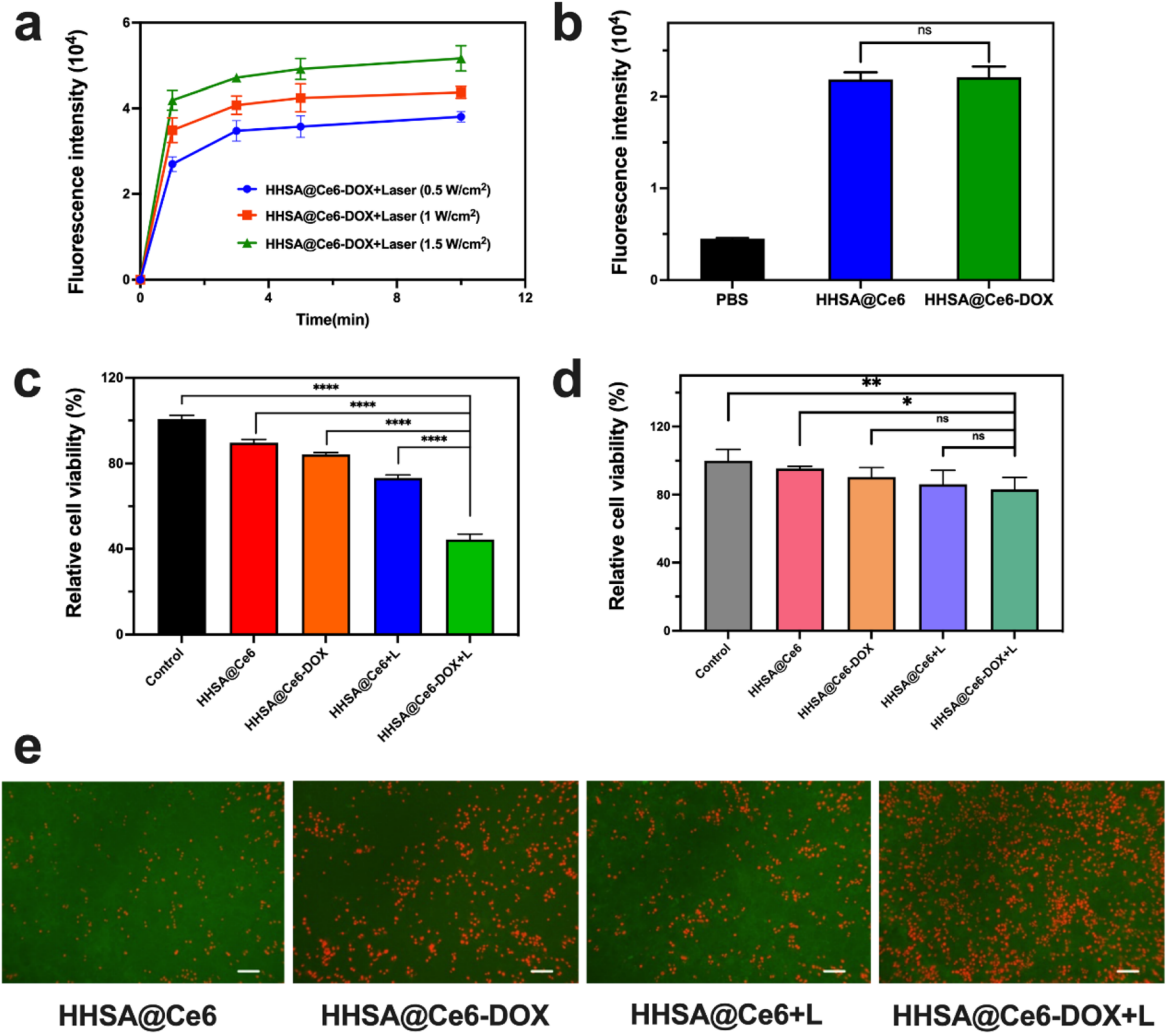
(a) The fluorescence intensity changes of SOSG with different times between different groups (Ce6 was set to 1 μg mL^−1^). (b) The fluorescence intensity of DCFH-DA in the HHSA@Ce6 and HHSA@Ce6-DOX groups (0.625 μg mL^−1^ Ce6 equiv.) in 4T1 cells. Relative cell viability of 4T1 cells (c) and 293T (d) cells before and after the irradiation (+L) in different treatment groups (0.625 μg mL^−1^ Ce6 equiv.). (e) Fluorescent images of live (green) and dead (red) cells before and after the irradiation (+L) in different treatment groups (0.625 μg mL^−1^ Ce6 equiv.) in 4T1 cells. Scale bar: 100 μm. *P* values: ns *P* > 0.05, *P < 0.05, **P < 0.01, ***P < 0.001, ****P < 0.0001 were calculated by SPSS with a *t*-test.

The SOSG probe was used to evaluate the ROS generation of HHSA@Ce6-DOX under irradiation with different power and at different times. Fig. 5a showed that the HHSA@Ce6-DOX can produce a certain amount of ROS after 660 nm laser irradiation, and the ROS production increased with time. When the laser power increased, HHSA@Ce6-DOX could generate more ROS at the same illumination time.

### 3.5 *In vitro* PDT

Next, we compared the photodynamic therapeutic effect of HHSA@Ce6 and HHSA@Ce6-DOX. 4T1 cells were irradiated with a 660 nm laser for 5 min (1 W cm^−2^), and then detected with MTT after incubated with HHSA@Ce6 and HHSA@Ce6-DOX (0.625 μg mL^−1^ Ce6 equiv.) for 24 h, respectively. The results showed that the HHSA@Ce6-DOX nanocapsules could effectively kill tumor cells under laser (Fig. 5c) and the curative effect of HHSA@Ce6-DOX was better than that of HHSA@Ce6. Compared to the HHSA@Ce6 group, the relative cell activity in the HHSA@Ce6-DOX group decreased from 74% to 45%. This was due to tumor cells being simultaneously killed by Ce6-triggered PDT and DOX-brought chemotherapy in the HHSA@Ce6-DOX therapeutic system.^18^

Meanwhile, we used 293T cells to verify the killing effect of HHSA@Ce6-DOX on normal human cells. The results showed that the relative survival rate of 293T cells remained above 80% with or without laser irradiation, which implied that HHSA@Ce6-DOX had no obvious cytotoxicity to human and embryonic kidney 293T cells (Fig. 5d). Under the same conditions, the survival rates of 293T cells in the HHSA@Ce6-DOX groups were slightly lower than those of HHSA@Ce6, which may be the slight damage of DOX to cells.

The intracellular ROS was detected *via* DCFH-DA probe in the HHSA@Ce6 and HHSA@Ce6-DOX (0.625 μg mL^−1^ Ce6 equiv.) groups to further evaluate the mechanism of HHSA@Ce6-DOX. DCFH-DA without fluorescence can be hydrolyzed into DCFH by intracellular esterase after entering the cell. While DCFH cannot penetrate the cell membrane and remain in the cells, the ROS in the cells oxidizes the non-fluorescent DCFH to generate DCF with fluorescence. The amount of intracellular ROS is reflected by detecting the fluorescence intensity of DCF. The results showed that the fluorescence intensity of HHSA@Ce6 and HHSA@Ce6-DOX loading Ce6 was much higher than that of PBS groups, indicating that Ce6 uptaking into the cells could effectively generate ROS under the 660 nm laser (Fig. 5b). And there was no statistical difference between HHSA@Ce6 and HHSA@Ce6-DOX groups due to the same concentration of Ce6. There is no significant difference in the amount of ROS produced by the two groups. According to Fig. 5c, the relative cell viability of HHSA@Ce6-DOX group is lower than that of HHSA@Ce6 group, indicating that DOX loaded in HHSA@Ce6-DOX nanocapsules can further kill tumor cells through chemotherapy, and the synergistic chemotherapy and PDT can kill tumors more effectively. To more intuitively compare the cell killing ability of individual and synergistic therapy, the live/dead staining of 4T1 cells in different treatment groups was observed by fluorescence microscope (Fig. 5e). Fluorescent images demonstrated that a large amount of red fluorescence representing dead cells was shown in HHSA@Ce6-DOX group, HHSA@Ce6 the green fluorescence representing live cells was much more than that in HHSA@Ce6-DOX group. This further showed that the improvement of anti-tumor effect in synergistic photodynamic and chemotherapy.

### 3.6 *In vivo* biocompatibility

**Fig. 6:**
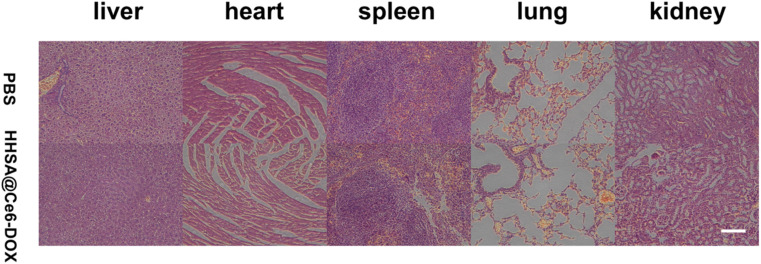
H&E-stained major organs of mice administered with PBS and HHSA@Ce6-DOX (10 μg mL^−1^ Ce6 equiv.). Scale bars: 100 μm.

To evaluate the biological toxicity, major organs hematoxylin and eosin (H&E) staining were performed for the mice administered with PBS and HHSA@Ce6-DOX (10 μg mL^−1^ Ce6 equiv.). The HHSA@Ce6-DOX group and the PBS group both did not show pathological changes in the heart, liver, spleen, lungs, and kidneys (Fig. 6) which reflected that HHSA@Ce6-DOX had the low biological toxicity and was a relatively secure therapeutic agent.

### 3.7 The photodynamic/chemo therapy *in vivo*

**Fig. 7:**
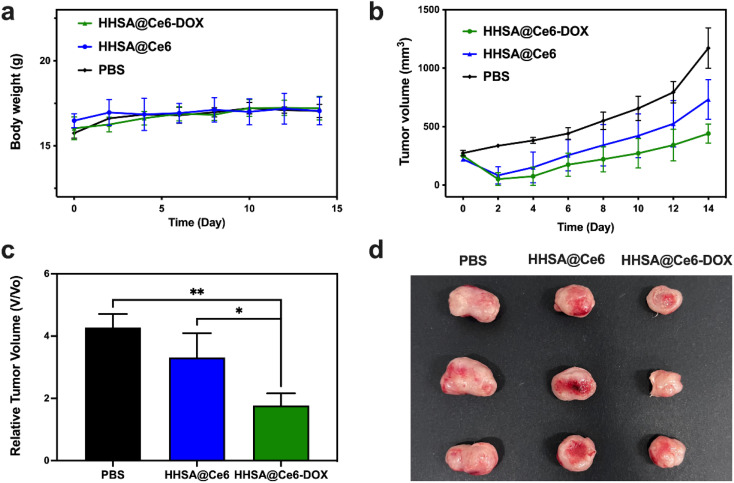
Body weight (a) and tumor volumes (b) of the mice with 4T1 tumors in different treatment groups with the same Ce6 concentration (PBS, HHSA@Ce6, HHSA@Ce6-DOX) within 14 days. (c) Relative tumor volume on the 14th day. (d) The photograph of tumors in different treatment groups on the 14th day. *P* values: ns *P* > 0.05, *P < 0.05, **P < 0.01 were calculated by SPSS with a *t*-test.

After confirming the good biocompatibility, the photodynamic/chemo efficacy of HHSA/CAT@Ce6 was assessed *in vivo*. The body weight and tumor volume of the mice treated with different methods were measured and recorded within 14 days. Fig. 7a showed that the weight of mice is relatively stable within 14 days after treatment, which indicated that these nanomaterials have no obvious biological toxicity. Fig. 7b–d presented the changes in tumor volumes of different treatment groups within 14 days. The volume of 4T1 tumor in the PBS group continued to increase rapidly, while the growth of 4T1 tumor in the treatment group injected with nanomaterials was inhibited. The result indicated that free laser had no significant therapeutic effect on tumors, while PDT could kill tumor cells to a certain extent. The tumor volumes in the HHSA@Ce6-DOX group were smaller than those of the HHSA@Ce6 group, which confirmed that DOX-mediated chemotherapy further inhibited tumor growth. The curative effect of the combination of chemotherapy and PDT was better than that of the single PDT.

## 4. Discussion

Due to the short blood circulation time and poor tumor targeting of conventional PSs, flexible nanoplatform HHSA based on HSA was synthesized through a hard core assisted template method to improve the transportation and uptake efficiency of anti-cancer drugs for tumor cells. The existing papers have confirmed that flexible nanomaterials can successfully reduce the internalization of immune cells, prolong the blood circulation time, and improve the uptake efficiency of tumor cells on nanomaterials. HSA can actively target tumor cells through gp60 and SPARC receptors.

The characterization of HHSA showed that HHSA nanocapsules have uniform size, good dispersion, and a hollow structure. The results of this experiment indicated that more than 80% of the relatively cell vitality can be maintained after empty loaded HHSA nanocapsules or HHSA@Ce6 were cultured with 4T1 cells for 24 h. In addition, no significant pathological damage was found in the main organs of BALB/c mice after the injection of HHSA-based HHSA@Ce6-DOX nanocapsules into BALB/c mice, indicating that flexible HHSA nanomaterials had excellent biocompatibility because HSA is the most abundant plasma protein in the human body.

After the chemotherapy drug DOX was loaded on the HHSA nanocapsules, the nanocapsules without 660 nm irradiation could kill some tumor cells and the effect of HHSA@Ce6-DOX was slightly better than that of HHSA@DOX. This may be that the π–π stacking force and hydrophobic interaction between hydrophobic Ce6 and DOX increase the loading amount of DOX. Compared with HHSA@Ce6, HHSA@Ce6-DOX can make the killing quantity of tumor cells increase from 30% to 55% due to the synergistic chemotherapy. DCFH-DA was used to detect ROS produced by the two kinds of nanocapsules based on HHSA at the cellular level. There was no statistical difference between the HHSA@Ce6 and HHSA@Ce6-DOX groups, further indicating that the PDT efficacy of the two groups was similar when the concentration of Ce6 was equal and DOX-mediated chemotherapy additionally increased the mortality of tumor cells.

## 5. Conclusion

Flexible HHSA nanocapsules were successfully synthesized by core-assisted protein-coating route. HHSA nanocapsules not only can lengthen blood circulation time *via* good dispersibility, but also can increase the uptake of HHSA through targeting gp60 and SPARC receptors and unique flexible hollow structures to enhance the efficiency of HHSA as drug delivery carriers. The results of experiments *in vitro* showed that, compared with individual chemotherapy or PDT nanoplatforms, HHSA@Ce6-DOX had a better anticancer effect *via* the combination PDT with chemotherapy after loading photosensitizer Ce6 and anticancer drug DOX. This design provides a new idea for the optimization of other anti-tumor therapies.

## Ethical statement

The animal study was reviewed and approved by The Animal Ethics Committee of Nanjing First Hospital (DWSY-2101557).

## Conflicts of interest

The authors declare that they have no known competing financial interests or personal relationships that could have appeared to influence the work reported in this article.

## Supplementary Material
